# Hospital-acquired bloodstream infections in patients deceased with COVID-19 in Italy (2020–2021)

**DOI:** 10.3389/fmed.2022.1041668

**Published:** 2022-11-17

**Authors:** Monica Monaco, Marco Floridia, Marina Giuliano, Luigi Palmieri, Cinzia Lo Noce, Annalisa Pantosti, Anna Teresa Palamara, Silvio Brusaferro, Graziano Onder, The Italian National Institute of Health COVID-19 Mortality Group, Luigi Palmieri

**Affiliations:** ^1^Department of Infectious Diseases, Istituto Superiore di Sanità, Rome, Italy; ^2^National Center for Global Health, Istituto Superiore di Sanità, Rome, Italy; ^3^Department of Cardiovascular, Endocrine-Metabolic Diseases and Aging, Istituto Superiore di Sanità, Rome, Italy; ^4^Office of the President, Istituto Superiore di Sanità, Rome, Italy

**Keywords:** COVID-19, secondary infections, bloodstream infections, bacterial infections, fungal infections, antimicrobial resistance

## Abstract

**Introduction:**

In hospitalized patients with COVID-19, bloodstream infections (BSI) are associated with high mortality and high antibiotic resistance rates. The aim of this study was to describe BSI etiology, antimicrobial resistance profile and risk factors in a sample of patients deceased with COVID-19 from the Italian National COVID-19 surveillance.

**Methods:**

Hospital charts of patients who developed BSI during hospitalization were reviewed to describe the causative microorganisms and their antimicrobial susceptibility profiles. Risk factors were analyzed in univariate and multivariate analyses.

**Results:**

The study included 73 patients (71.2% male, median age 70): 40 of them (54.8%) received antibiotics and 30 (41.1%) systemic steroids within 48 h after admission; 53 (72.6%) were admitted to intensive care unit. Early steroid use was associated with a significantly shorter interval between admission and BSI occurrence. Among 107 isolated microorganisms, the most frequent were *Enterococcus* spp., *Candida* spp., *Acinetobacter baumannii*, and *Klebsiella pneumoniae*. Median time from admission to BSI was shorter for *Staphylococcus aureus* compared to all other bacteria (8 vs. 24 days, *p* = 0.003), and longer for *Enterococcus* spp., compared to all other bacteria (26 vs. 18 days, *p* = 0.009). Susceptibility tests showed a high rate of resistance, with 37.6% of the bacterial isolates resistant to key antibiotics. Resistance was associated with geographical area [adjusted odds ratio (AOR) for Central/South Italy compared to North Italy: 6.775, *p* = 0.002], and with early use of systemic steroids (AOR 6.971, *p* = 0.018).

**Conclusions:**

In patients deceased with COVID-19, a large proportion of BSI are caused by antibiotic-resistant bacteria. Early steroid use may facilitate this occurrence.

## Introduction

Several studies, systematic reviews and metanalyses have evaluated prevalence, microbiological characteristics, clinical course and outcomes of secondary infections in patients hospitalized with COVID-19 (1–8).

Most of these studies have indicated a high incidence of secondary infections, usually defined by positive culture of a new pathogen ≥ 48 h after hospital admission (9) in patients with COVID-19, that also appear to be associated with a more severe course of the disease and with increased mortality (3–5, 10). Pasquini et al. report an incidence rate of 8.19 episodes of bloodstream infections (BSI) per 1,000 patient-days among patients with COVID-19, compared to 2.72 in patients without COVID-19, and thirty-day mortality rates for BSI of 40.2 and 23.7% in patients with and without COVID-19, respectively (1). In the study by Afzal et al. the incidence of BSI was 4.37 per 1,000 patient-days in the pre-COVID-19 period compared to 8.36 during the surge (2). Even higher rates have been described in patients hospitalized in intensive care units (ICU) (11.7 BSI episodes per 1,000 patient-days) (10) or in critically ill patients (47/1,000 patients-days) (4). Ippolito et al. in a systematic review and meta-analysis estimated at 7.3% the occurrence of BSI among hospitalized patients with COVID-19, corresponding to a 2.77-fold risk increase compared to patients without COVID-19; the occurrence of BSI was remarkably higher in COVID-19 patients admitted to ICU (29%). In the same analysis, patients hospitalized with COVID-19 complicated by BSI had an estimated mortality rate of 41% (5).

Other studies have addressed the prevalence and characteristics of antimicrobial resistance in patients with COVID-19, suggesting increased rates of drug resistance and different antimicrobial resistance profile compared to the pre-COVID-19 era or to non-COVID-19 populations (3, 8, 11–13). Baker et al. reported that COVID-19 surges were significantly associated with increases in rates of hospital-onset bloodstream infections and multidrug resistant organisms, including methicillin-resistant *Staphylococcus aureus* (MRSA), vancomycin-resistant *Enterococcus* spp. (VRE), and Gram-negative organisms (3). Other studies reported a high prevalence of *Acinetobacter baumannii* in COVID-19 associated BSIs (8, 12, 13). A systematic review and meta-analysis showed a high prevalence of antimicrobial resistance (AMR) in COVID-19 patients, with the most common microorganisms represented by MRSA, carbapenem-resistant *A. baumannii, Klebsiella pneumonia*e and *Pseudomonas aeruginosa*, and multi-drug resistant *Candida auris* (11). High rates of carbapenem resistance in *Acinetobacter* spp., *K. pneumoniae* and other Enterobacterales have also been reported in single studies where the above microorganisms represented the predominant causes of BSI in patients hospitalized with COVID-19 (12, 13). Both prevalence of BSI and rates of resistance, however, displayed wide heterogeneity by hospital and geographical area (8–13). Since Italy is the European country with the estimated highest number of antibiotic-resistant infections and associated deaths (14), it is necessary to collect updated information on the microbiological profile of BSI and on possible determinants of antimicrobial resistance in patients with COVID-19, in order to identify risk factors, improve outcomes, and reduce the occurrence of antimicrobial resistance.

In order to explore this issue, we expanded our previous investigations in patients deceased with COVID-19 (15), with the intent of focusing on the occurrence of bloodstream infections (BSI) after hospitalization for COVID-19. The aim of this analysis was to define the general characteristics of BSI, including causative pathogens, time between admission and occurrence, time between occurrence and death, occurrence in intensive care unit (ICU) or in other wards, and to identify possible risk factors for more rapid development of BSI. Additional objectives were represented by the description of the antimicrobial resistance profiles of the involved microorganisms and by the identification of possible determinants of antimicrobial resistance.

## Materials and methods

### Study population

The present study was nested in the Italian National COVID-19 Surveillance on causes of death in individuals with SARS-CoV-2 infection, coordinated by the Istituto Superiore di Sanità (ISS, the Italian National Institute of Health). Within this surveillance, all Italian Regions and Autonomous Provinces send to ISS the hospital records of patients with PCR-confirmed SARS-CoV-2 infection who deceased in hospital. At ISS, a random sample of such records is periodically assigned to clinical review, ensuring that the proportion of hospital records to be reviewed for each region matches the proportion of deaths per region observed within the same period. Key data from the records (demographics, comorbidities, admission to ICU, time interval from hospitalization to death, occurrence of complications, and cause of death as reported on the official death certificate) are reviewed by a team of medical doctors at ISS and entered into a surveillance database. We here analyzed a sample of 157 clinical records from this database already described in a previous paper (15), for BSI characteristics and correlates. For the present study, hospital charts, documenting the diagnosis of BSI, were further reviewed by two of the authors, who extracted additional information on early (within 48 h from admission) treatment with antibiotics and steroids, time and type of clinical samples collected for microbiological investigations, detected microorganisms, antimicrobial resistance profiles, time from hospitalization and from ICU admission to development of secondary infections. Only systemic steroids were considered.

COVID-19 patients admitted to hospital from February 2020 to April 2021, roughly corresponding to the first (February 2020–September 2020) and second wave (October 2020–April 2021) of the COVID-19 pandemic in Italy (16) were included. The main intent of the study was descriptive.

### Definitions and categorization of bloodstream infections

Eligibility criteria for this analysis were represented by the presence of microbiologically confirmed hospital-acquired BSI, defined as positive blood cultures for bacteria or fungi from peripheral blood, central or peripheral venous catheter obtained after 48 h from hospital admission. Presence of common skin contaminants, such as coagulase-negative *Staphylococcus* spp., *Corynebacterium* spp. and *Bacillu*s spp., was not considered. Blood cultures were taken upon clinical suspicion (e. g., in presence of fever ≥ 38°C).

BSI were categorized by detected microorganism, ward of occurrence (ICU or other clinical departments), and source of the sample [peripheral blood, central venous catheter (CVC) or peripheral venous catheter (PVC)]. For antibiotic resistance, we focused on the microorganisms considered as critical or high priority by WHO, with the corresponding most relevant antibiotic resistance traits (17). More specifically, the drug-resistant organisms analyzed were: carbapenem-resistant (CR) *Acinetobacter baumannii*; CR-*Pseudomonas aeruginosa*; CR and/or 3rd generation cephalosporin resistant (3GCR) *K. pneumoniae* and other Enterobacterales, vancomycin-resistant *Enterococcus faecium* or *Enterococcus faecalis* (VRE), and methicillin-resistant *Staphylococcus aureus* (MRSA) (17).

### Statistical analysis

Quantitative variables were compared using the Mann–Whitney U-test or Student T test (unadjusted for multiple comparisons), and categorical variables with the chi-square test or the Fisher test, as appropriate. The independent role of some potentially relevant covariates on time interval between admission and a positive blood culture was analyzed in a multivariate linear regression model that used time interval between admission and first positive culture as the dependent variable. The independent association of different covariates with presence of resistance was analyzed in a multivariable logistic regression model, and adjusted odds ratios (AOR) with 95% confidence intervals (CI) were calculated. The inclusion of covariates in the multivariable models was selected based on level of significance < 0.10 in univariate analyses, possible direct causative role, and improvement in the fitness of the model in terms of higher values of R^2^.

For all analyses *P*-values < 0.05 were considered statistically significant. All analyses were performed using the SPSS software, version 27.0 (IBM Corp, 2017, Armonk, NY, USA).

## Results

The initial sample previously described included 157 patients. Following exclusion of 73 patients who had only respiratory infections and of 11 additional patients with BSI occurring within 48 h from admission, the study included 73 patients with hospital-acquired BSI. Their general characteristics are reported in Table 1. Median age was 70 years (IQR 60–79), Most of the patients (71.2%) were males, were coming from their homes at admission (76.7%), and 61.6% lived in North Italy. In the first 48 h from admission 40 patients (54.8%) received antibiotics, and 30 (41.1%) received systemic steroids.

**TABLE 1:** Population characteristics.

Number of evaluated patients	73	
	*N*	%
Age:		
<65 years	46	63.0
≥65 years	27	37.0
Sex:		
Male	52	71.2
Female	21	28.8
Patient location before admission:		
Home	56	76.7
Transferred from another hospital or from a long-term facility	17	23.3
Geographical area (Italy):		
North	45	61.6
Center/south	28	38.4
Period of hospitalization:		
February 2020–September 2020	34	46.6
October 2020–April 2021	39	53.4
Comorbidities:		
Chronic respiratory disease	16	21.9
Neoplastic disease	9	12.3
Diabetes	18	24.7
Chronic renal failure	13	17.8
Antibiotics in the first 48 h from admission:		
Any	40	54.8
Azithromycin	18	24.7
Ceftriaxone	17	23.3
Piperacillin-tazobactam	11	15.1
Others	10	13.7
Systemic steroids in the first 48 h from admission	30	41.1
Number of microorganisms isolated from blood cultures during hospitalization:		
One, single episode/culture	45	61.6
Two, single episode/culture	6	8.2
Two, different episodes/cultures	17	23.3
Three, different episodes/cultures	4	5.5
Four, different episodes/cultures	1	1.4
Admitted to Intensive Care Unit (ICU) during hospitalization	53	72.6
Sepsis reported as a cause of death in the death certificate	36	49.3
Days from hospital admission to ICU admission (median, IQR)	3 (0–8)	
Days from hospital admission to first positive blood culture (median, IQR)	20 (13–29.5)	
Days from ICU admission to first positive blood culture (median, IQR)	14 (8–20.5)	
Days from hospital admission to death (median, IQR)	35 (21–59)	
Days from ICU admission to death (median, IQR)	34 (16–50)	

IQR, interquartile range.

The majority of patients (69.8%) had a single positive blood culture, with one (61.6%) or, rarely, two (8.2%) isolated microorganisms. Fifty-three patients (72.6%) were admitted to the ICU, after a median interval of 3 days from hospital admission. First positive blood culture occurred after a median interval of 20 days after hospital admission, and death after a median interval of 35 days from hospital admission (Table 1).

The time interval (days) between admission and first positive blood culture was analyzed according to different characteristics at admission (Table 2).

**TABLE 2:** Time from hospital admission to first positive blood culture according to different potentially relevant covariates.

Covariate	Time (days) from hospital admission to first positive blood culture: median (IQR)	*P*-value
Age < 65 (n: 27)	21 (16-30)	0.092
Age ≥ 65 (n: 46)	19 (8-28)	

ICU (n: 53)	20 (13-28)	0.420
Other ward (n: 20)	21 (14-32)	

Use of systemic steroids < 48 h (n: 30)	16 (8-24)	0.018
No use of systemic steroids < 48 h (n: 43)	21 (16-31)	

Use of antibiotics < 48 h (n: 40)	20 (14-30)	0.527
No use of antibiotics < 48 h (n: 33)	20 (11-28)	

Male (n: 52)	20 (12-28)	0.213
Female (n: 21)	24 (16-35)	

North Italy (n: 45)	20 (13-29)	0.540
Center/South Italy (n: 28)	18 (12-29)	

February 2020–September 2020 (n: 34)	21 (14-32)	0.211
October 2020–April 2021 (n: 39)	19 (11-26)	

Chronic respiratory diseases (n: 16)	21 (10-29)	0.968
No chronic respiratory diseases (n: 57)	20 (13-30)	

Neoplastic diseases (n: 9)	21 (17-24)	0.827
No neoplastic diseases (n: 64)	20 (13-30)	

Diabetes (n: 18)	17 (11-30)	0.599
No diabetes (n: 55)	20 (14-29)	

Chronic renal failure (n: 13)	17 (8-26)	0.387
No chronic renal failure (n: 60)	20 (14-30)	

Coming from home (n: 56)	20 (13-28)	0.749
Institutionalized/From other hospital (n: 17)	19 (11-31)	

Any main comorbidity* (n: 41)	20 (10-28)	0.552
No comorbidities (n: 32)	20 (5-30)	

*Diabetes, chronic respiratory diseases, renal failure, neoplastic diseases. Chronic renal failure was defined as the presence of a GFR < 60 ml/min for at least 3 months. Chronic respiratory diseases included chronic obstructive pulmonary disease, and/or use of domiciliary oxygen therapy.

Use of systemic steroids within the first 48 h was associated with a significantly shorter interval between admission and first positive blood culture (16 vs. 21 days, *p* = 0.018). No significant differences were found for the remaining considered variables. Older age was associated with a slightly shorter time interval, but the difference did not reach statistical significance (19 vs. 21 days, *p* = 0.092). In order to adjust for covariates, we evaluated the independent role of different potentially relevant covariates in a multivariate linear regression model that used the time interval between admission and first positive culture as the dependent variable. The results of this analysis are presented in Table 3.

**TABLE 3:** Multivariate linear regression model of factors affecting time from hospital admission to first positive blood culture.

Covariate	B coefficient	B coefficient, 95% CI		*P*-value
		Lower limit	Upper limit	
Age ≥ 65	–6.193	–12.909	0.524	0.070
Systemic steroids within 48 h from admission	–9.552	–15.973	–3.131	0.004
ICU	–2.672	–10.114	4.770	0.476
Antibiotics within 48 h from admission	5.268	–1.127	11.664	0.105
Male sex	–6.719	–13.609	0.171	0.056
Presence of comorbidities	–1.423	–7.462	4.615	0.639

CI, confidence interval; ICU, intensive care unit. Comorbidities: any of the following: diabetes, chronic respiratory diseases, renal failure, neoplastic diseases.

The significant association between use of corticosteroids within the first 48 h from admission and time to positive culture was confirmed after adjusting for covariates, with an estimated significantly shorter interval (9.5 days, 95%CI 3–15) associated with early use of systemic steroids. Effects approaching statistical significance were also found for male sex and older age (shorter interval) and for use of antibiotics within 48 h from admission (longer interval) (Table 3).

Overall, a total of 107 microorganisms were isolated from the evaluated patients. Their distribution according to source of sampling and to hospital ward is reported in Table 4.

**TABLE 4:** Microorganisms isolated by source of sample and hospital ward.

	All	Source of sample cultured		Hospital ward	
		Peripheral blood	CVC or PVC	ICU	Other
*Enterococcus* spp.	34	21 (61.8%)	13 (38.2%)	28 (82.4%)	6 (17.6%)
*Candida* spp.	16	14 (87.5%)	2 (12.5%)	8 (50.0%)	8 (50.0%)
*Acinetobacter baumannii*	14	5 (35.7%)	9 (64.3%)	14 (100.0%)	0
*Klebsiella pneumoniae*	12	7 (58.3%)	5 (41.7%)	9 (75.0%)	3 (25.0%)
*Staphylococcus aureus*	10	8 (80.0%)	2 (20.0%)	5 (50.0%)	5 (50.0%)
*Pseudomonas aeruginosa*	8	6 (75.0%)	2 (25.0%)	7 (87.5%)	1 (12.5%)
Other Enterobacterales	7	5 (71.4%)	2 (28.6%)	6 (85.7%)	1 (14.3%)
Other bacteria	6	5 (83.3%)	1 (16.7%)	4 (66.7%)	2 (33.3%)
All	107	71 (66.4%)	36 (33.6%)	81 (75.7%)	26 (24.3%)

CVC, central venous catheter; PVC, peripheral venous catheter; ICU, Intensive Care Unit.

The most frequently isolated pathogens were *Enterococcus* spp. (n: 34), *Candida* spp. (n: 16), *A. baumannii* (n: 14) and *K. pneumoniae* (n: 12). Most cultures were obtained from peripheral blood samples (66.4%) and during the stay of the patients in ICU (75.7%).

Time from admission to positive culture was not different for cultures obtained from peripheral blood or from venous catheters (20 days for both, *p* = 0.561). Table 5 illustrates the different time intervals between admission and positive culture and between positive culture and death for individual microbial pathogens.

**TABLE 5:** Time from admission to positive blood culture and time from positive blood culture to death, by isolated microorganism.

Microorganism	Time (days) from admission to positive blood culture: median (IQR)	Time (days) from positive blood culture to death: median (IQR)
*Enterococcus* spp. (n: 34)	26(20−39)*	12(5−43)
*Candida* spp. (n: 16)	27(13−35)	11(4−32)
*Acinetobacter baumannii* (n: 14)	19(11−27)	22(10−44)
*Klebsiella pneumoniae* (n: 12)	29(16−37)	13(5−42)
*Staphylococcus aureus* (n: 10)	8(5−16)*	10(8−17)
*Pseudomonas aeruginosa* (n: 8)	30(18−43)	14(10−29)
Other Enterobacterales (n: 7)	16(9−26)	9(4−19)
Other bacteria (n: 6)	17(5−37)	12(8−21)
All (n: 107)	23(14−35)	13(6−32)

*p < 0.01 (compared to all the other bacteria), Mann–Whitney U test.

In pairwise comparisons based on all the 91 bacterial isolates, median time from admission to positive blood culture was shorter for *S. aureus* (8 days, IQR 5–16) compared to all other bacterial microorganisms (24 days, IQR 16–36, *p* = 0.003), and longer for *Enterococcus* spp. (26 days, IQR 20–39) compared to all other bacteria (18 days, IQR 10–30, *p* = 0.009). No significant differences were found for the pairwise comparisons between *A. baumannii, K. pneumoniae* and *P. aeruginosa* and all other bacteria (*p*-values: 0.148, 0.313, and 0.190, respectively). The analysis of time between positive blood culture and death showed no significant differences among the bacterial species considered (all *p*-values > 0.1).

Susceptibility tests were available for 85/91 (93.4%) bacterial isolates and for 11/16 (68.7%) *Candida* spp. isolates. The most relevant antimicrobial resistance traits of the microorganisms isolated are summarized in Table 6.

**TABLE 6:** Pathogens isolated from blood cultures and resistance profile.

Pathogen	Resistant, *n* (%)
*Staphylococcus aureus* (n: 10)	6/10 (60%) MRSA
*Enterococcus* spp. (n: 32)	0/32 (0%) VRE
*Klebsiella pneumoniae* (n: 11)	7/11 (63%) CR 9/11 (81%) 3GCR
Other Enterobacterales (n: 7)	0/7 (0%) CR 3/7 (43%) 3GCR
*Pseudomonas aeruginosa* (n: 7)	1/7 (14.3%) CR
*Acinetobacter baumannii* (n: 13)	13 (100%) CR
Other bacteria (n: 5)*	0/5 (0%) CR, 3GCR
*Candida* spp. (n: 11)	1/11 (9%) Azole-resistant

MRSA, methicillin-resistant *S. aureus*; VRE, vancomycin-resistant *E. faecium* or *E. faecalis*; CR, carbapenem-resistant; 3GCR, 3rd-gen. cephalosporin-resistant. *Bacteroides fragilis (n: 2), *Burkholderia gladioli* (n: 1), *Sphingomonas mucosissima* (n: 1), *Streptococcus pneumoniae* (n: 1).

The overall rate of resistance to key antibiotics among bacterial isolates was 37.6% (32/85). Presence of antibiotic resistance in bacteria was not significantly associated with sex (38.7% in males, 34.8% in females, *p* = 0.740), patient location before admission (38.2% for home, 35.3% for hospital/institution, *p* = 0.823), presence of co-morbidities (41.3% compared to 33.3% for no morbidities, *p* = 0.450), first vs. second wave of the pandemics (34.9% vs. 40.5%, respectively; *p* = 0.595), use of antibiotics in the 48 h before admission (44.0% with antibiotics, 28.6% without antibiotics, *p* = 0.148), and hospital ward (40.8% for ICU, 21.4% for others, *p* = 0.171).

However, resistant bacteria were significantly more prevalent in patients with age < 65 years (51.4% vs. 27.1% in patients ≥ 65 years, *p* = 0.022), in patients hospitalized in Central-Southern Italy (56.4% vs. 21.7% in North Italy, *p* = 0.001), in blood cultures obtained from CVC or PVC (51.6%, compared to 29.6% in peripheral blood, *p* = 0.044), and in patients who had taken steroids in the 48 h before admission (51.4% vs. 28.0% with no steroids, *p* = 0.028).

The independent role of the above covariates on development of resistance was analyzed in a multivariable logistic regression model. After adjusting for entry in ICU, source of sample, antibiotic use in the first 48 h from admission, age, sex, wave of the pandemic, presence of comorbidities and admission from home or hospital/institution, presence of antimicrobial resistance remained associated with geographical area (adjusted odds ratio for Central/Southern Italy compared to North Italy: 6.775, 95%CI 2.005–22.982, *p* = 0.002), and with use of systemic steroids in the first 48 h from admission (adjusted odds ratio: 6.971, 95%CI 1.386–35.064, *p* = 0.018). No significant associations were found for the remaining considered covariates.

## Discussion

This study described the characteristics of BSI in a national series of patients deceased in hospital with COVID-19 infection. Several studies have indicated a higher incidence of BSI in COVID-19 patients compared to contemporary or historical non-COVID-19 populations (1, 2, 5, 18). According to a recent meta-analysis, the occurrence of BSI in hospitalized COVID-19 patients was 7.3% that increased to 29.6% in patients admitted to ICU (5). BSI in patients with COVID-19 are associated with higher mortality and shorter time to death (1, 7, 8, 13, 19, 20). In addition, higher antibiotic resistance rates have been documented in the BSI bacterial pathogens (3, 7).

In the analysis of relevant bacterial species, for their frequency, virulence and antibiotic resistance rates, we focused on microorganisms of the so called ESKAPE group (*Enterococcus* spp., *S. aureus, K. pneumoniae, A. baumannii, P. aeruginosa*, other Enterobacterales) (21). In our case series, *Enterococcus* spp. represented the most common bacterial cause of BSI. An increased prevalence of enterococcal BSI in patients with COVID-19 has been described (2, 4, 6, 10). Possible underlying mechanisms of this high occurrence include enterococcal colonization of the respiratory tract associated with intubation and traslocation of enteric bacteria through a damaged intestinal mucosa (2, 22). A frequent occurrence of BSI due to *A. baumannii* and *K. pneumoniae* has also been reported by others (7, 8, 13, 20).

Few studies have evaluated the time from hospital admission to BSI and its determinants in patients with COVID-19. In our study, the median interval from admission to BSI was 20 days, longer than the corresponding interval of 9 days reported by Massart et al. (20), and also longer than the entire course of hospitalization between admission and death (11 days) reported by Palanisamy et al. (8). Our data are more consistent with those by Pasquini et al. (1), who report a median interval of 16 days between hospitalization and BSI, although the same authors also found a short median interval of 5 days between BSI and death.

Although such variability may have several causes, we found some significant differences in time between hospitalization and occurrence of BSI by causative agent and, most importantly, by early use of systemic steroids after admission. These and other local determinants may explain differences among studies, suggesting that the involved factors may be multiple.

Median time between hospitalization and death was 35 days. We found no significant differences in this interval among microorganisms. However, it should be underlined that we were unable to evaluate the outcome of BSI and its possible resolution, and we have only limited information on the contributing role of the infection in the patients’ death. An analysis of the death certificates showed that sepsis was reported as a factor contributing to death, for half of the patients, indirectly confirming the association between BSI and mortality already described in these patients.

We also analyzed rate and determinants of antimicrobial resistance. In a recent systematic review and meta-analysis of antimicrobial resistance in patients with COVID-19, 29% of the identified microorganisms were resistant to one or more antibiotics (11). Our rate of 37.6% for antibiotic resistance to critical antibiotics appears considerably high. This difference may be due to differences among the populations evaluated, that in our case included only patients with severe disease who had an unfavorable outcome and died in hospital. Similarly high rates of antibiotic resistance have been reported in studies where mortality was high (7, 8, 13). With respect to individual causative pathogens, our findings of universal (100%) resistance in isolates of *A. baumannii* and of sixty per cent resistance rate in *S. aureus* and *K. pneumoniae*, confirms similar previous findings of other studies conducted in COVID-19 patients in different geographical areas (7, 10, 13). With respect to fungal isolates, our results are consistent with those of Karuna et al., that describe a low rate of azole resistance in *Candida* spp. isolated from BSI (12).

In examining determinants of antimicrobial resistance, we found an association between increased risk of antimicrobial resistance and geographical area. Such data confirm that occurrence of BSI and rates of antimicrobial resistance are subject to geographical variability not only between countries but also within countries. In Italy, the antibiotic resistance rate in critical pathogens causing BSI is one of the highest in Europe, but there is large variation among the Italian regions (1, 23, 24). Such variability may be due to differential use of antibiotics and to other factors that have been broadly defined as “local ecology” (1).

Among potentially relevant factors, we could not confirm ICU admission as a factor significantly contributing to resistance (11), but the rate of antimicrobial resistance in ICU was almost double compared to other wards. Larger studies might confirm the significancy of this effect. Early use of antibiotics (at admission and in the first 2 days after admission) was not associated with the development of antibiotic resistance, although overuse of antibiotics in COVID-19 patients is often considered responsible for this increase (25).

Early (in the first 48 h from admission) use of systemic corticosteroids was associated with a shorter interval between admission and occurrence of BSI, and with a significantly higher risk of antimicrobial resistance. These findings are consistent with the increased risk of secondary infections described in patients with COVID-19 receiving corticosteroid treatment (4, 26, 27). This increased susceptibility to infections may, however, not necessarily translate in increased mortality: use of steroids might have beneficial effects in patients with ARDS, with and without COVID-19 (28), and may actually reduce mortality in patients with critical illness (29, 30). The shorter time to infection associated with corticosteroid use and the increased risk of antimicrobial resistance should therefore be weighed against the possible clinical benefits of treatment, particularly in more severe patients.

Our results would indirectly support the current guidelines for COVID-19 treatment which do not recommend the standardized use of steroids in non-hospitalized or hospitalized patients in the absence of another indication (31) or unless severe disease is present and oxygen supplementation is required (32). Such recommendations are mainly based on the findings of a large trial that indicated benefit of dexamethasone only for severely ill hospitalized COVID-19 patients, with potential harm in those not requiring oxygen (33).

Our study has some limitations that should be considered. The retrospective design may have introduced some selection bias, and the relatively low patient number may have hindered the detection of some significant associations that would have required a larger sample size. Moreover, the retrospective collection of the case series through hospital records has precluded a predefined harmonization of diagnostic procedures, sampling protocols, laboratory methods, and treatment administered across different centers. For systemic steroids, we did not collect information on the specific steroids that were used, and on their dosage. However, the inclusion of patients’ records from multiple centers has overcome the local factors that often determine important differences in the incidence and characteristics of BSI and in the antimicrobial resistance profiles (1, 7).

## Conclusion

We described the clinical and microbiological profiles of BSI in a multicenter study of patients with severe COVID-19 disease, providing new information. Some determinants of more rapid occurrence of BSI and of development of antimicrobial resistance were identified. Use of systemic corticosteroids appeared to influence significantly both the time between hospital admission and BSI and the risk of antimicrobial resistance. The findings confirm the need to avoid routine use of corticosteroids in COVID-19 patients unless precise clinical indications are present. A rational targeted use of antibiotics could also reduce the emergence and circulation of antibiotic-resistant strains that currently cause BSI in hospitalized patients with COVID-19, especially in countries as Italy where AMR is largely spread.

## The Italian National Institute of Health COVID-19 Mortality Group

Luigi Palmieri, Elvira Agazio, Pierfrancesco Barbariol, Antonino Bella, Eva Benelli, Luigi Bertinato, Matilde Bocci, Stefano Boros, Marco Bressi, Giovanni Calcagnini, Federica Censi, Alessandra Ciervo, Elisa Colaizzo, Cecilia Damiano, Martina Del Manso, Corrado Di Benedetto, Chiara Donfrancesco, Massimo Fabiani, Francesco Facchiano, Marco Floridia, Fabio Galati, Marina Giuliano, Tiziana Grisetti, Cecilia Guastadisegni, Cinzia Lo Noce, Pietro Maiozzi, Valerio Manno, Margherita Martini, Alberto Mateo Urdiales, Eugenio Mattei, Claudia Meduri, Paola Meli, Giada Minelli, Graziano Onder, Daniele Petrone, Patrizio Pezzotti, Flavia Pricci, Ornella Punzo, Flavia Riccardo, Chiara Sacco, Paolo Salerno, Debora Serra, Matteo Spuri, Marco Tallon, Manuela Tamburo De Bella, Dorina Tiple, Brigid Unim, Luana Vaianella, Maria Fenicia Vescio, Liliana Elena Weimer, and Silvio Brusaferro.

## Data availability statement

The datasets generated and analyzed during the current study are not publicly available due to confidentiality issues but are available from the corresponding author upon reasonable request.

## Ethics statement

The collection and scientific dissemination of data related to COVID-19 epidemics by the ISS and other public health bodies was authorized by law on February 27th, 2020, by the Italian Presidency of the Council of Ministers. Gazzetta Ufficiale Della Repubblica Italiana, Serie Generale, Parte Prima, February 28, 2020. Available online at: https://www.gazzettaufficiale.it/eli/gu/2020/02/28/50/sg/pdf (accessed October 10, 2022).

## Author contributions

MF, MG, AP, and MM conceived and designed the study and drafted and finalized the manuscript. MF, MG, and LP were responsible for statistical analysis. MF, MG, CLN, LP, GO, ATP, SB, and The Italian National Institute of Health COVID-19 Mortality Group substantially contributed to acquisition of data and critical revision of the manuscript. All authors approved the final version to be published.
